# Strategies for improving the 3D printability of decellularized extracellular matrix bioink

**DOI:** 10.7150/thno.81785

**Published:** 2023-04-23

**Authors:** Huihui Zhang, Yilin Wang, Zijun Zheng, Xuerong Wei, Lianglong Chen, Yaobin Wu, Wenhua Huang, Lei Yang

**Affiliations:** 1 Department of Burns, Nanfang Hospital, Southern Medical University, Jingxi Street, Baiyun District, Guangzhou, 510515, PR China; 2 Guangdong Engineering Research Center for Translation of Medical 3D Printing Application, Guangdong Provincial Key Laboratory of Medical Biomechanics, Department of Human Anatomy, School of Basic Medical Sciences, Southern Medical University, Guangzhou, China; 3 Guangdong Medical Innovation Platform for Translation of 3D Printing Application, Southern Medical University, The Third Affiliated Hospital of Southern Medical University, Southern Medical University, Guangzhou, China

**Keywords:** 3D bioprinting, bioink, microenvironment, tissue regeneration, decellularized extracellular matrix

## Abstract

3D bioprinting is a revolutionary technology capable of replicating native tissue and organ microenvironments by precisely placing cells into 3D structures using bioinks. However, acquiring the ideal bioink to manufacture biomimetic constructs is challenging. A natural extracellular matrix (ECM) is an organ-specific material that provides physical, chemical, biological, and mechanical cues that are hard to mimic using a small number of components. Organ-derived decellularized ECM (dECM) bioink is revolutionary and has optimal biomimetic properties. However, dECM is always "non-printable" owing to its poor mechanical properties. Recent studies have focused on strategies to improve the 3D printability of dECM bioink. In this review, we highlight the decellularization methods and procedures used to produce these bioinks, effective methods to improve their printability, and recent advances in tissue regeneration using dECM-based bioinks. Finally, we discuss the challenges associated with manufacturing dECM bioinks and their potential large-scale applications.

## Introduction

The growing demand for organ and tissue regeneration in patients is complicated by the dearth of suitable donors and concerns about immune rejection or biocompatibility after transplantation. Tissue engineering (TE) provides excellent prospects for overcoming the limitations of current therapeutic and organ transplantation methods 1. TE research attempts to generate biological alternatives for native human tissues to replace or repair damaged tissue, accomplish functional and structural tissue formation upon implantation, and mitigate current organ shortages and requirements for in vivo transplantation 2. In TE, biomaterials exhibit substantial superiority and strength 3.

3D bioprinting (3DBP) is an additive manufacturing process for generating hierarchical scaffolds with customized geometries and structures via the placement of bioink containing living cells and biomaterials, allowing the production of patient-specific implants and structures 4, 5. The scaffold directs the growth of cells in three dimensions during tissue development 6. Compared to conventional manufacturing approaches, 3DBP may be capable of delivering a construct with a pre-designed microstructure and cell arrangement for the scaffold and attaining structural, morphological, and mechanical diversity in the printed structure. The bioprinted scaffold provides a 3D culture environment for seeded cells by enhancing cell contact, cell-cell interactions, and cell-matrix interactions 7. Moreover, the 3D-printed tissue would be highly repeatable, which is essential for commercial production 8. However, reproducing the characteristics of the native extracellular matrix (ECM) is challenging. These characteristics include surface structure, mechanical properties, pore size, biocompatibility, biodegradability, and cellular adhesion 9. Natural or synthetic biomaterials cannot perfectly mimic the intricacy of the natural ECM and are therefore insufficient for replicating the milieu of live tissues.

The ECM mediates extracellular signaling to resident cells via its unique tissue-specific structure and protein composition 10. The cell-ECM interactions regulate tissue homeostasis, such as cell proliferation, migration, differentiation, and the neo-ECM formation process 9. Decellularized ECM (dECM) biomaterials have the potential to stimulate tissue regeneration by providing a native-like milieu 11. During decellularization, cells and immunogenic molecules are largely eliminated, whereas various functional and structural components, including glycosaminoglycans (GAGs), glycoproteins, and cytokines, are mostly preserved 12. 3DBP can integrate the biological qualities of the ECM with tunable morphological and mechanical characteristics 13, 14. dECM bioink can be placed in a pattern according to the size and structure of a defect or organ 15. For instance, bioprinted dECM scaffolds may comprise distinct layers and tissue-specific cells 16. The multilayer dECM scaffolds could provide cues to encapsulated cells and the surrounding environment to mimic the tissue's layered structure. Therefore, dECM has a promising potential in functional 3D-printed bioinks 17.

However, it is difficult to employ dECM alone as a bioink for 3D printing due to its low viscosity and mechanical instability 18. Hydrogels created with solubilized dECM are, at best, only slightly stiffer than those of pure collagen gels 19, with gelation periods ranging from 30 min to 1 hour 20. dECM is usually considered “non-printable” based on extrusion-based printing technology 21. However, 3D printing is expected to provide a physically robust structure with a high degree of shape integrity for computer-aided design. As a result, attempts have been made to enhance the printability of dECM bioinks and the stability of printed scaffolds by integrating them with polymer frameworks 22, mixing them with synthetic polymers, and employing crosslinkers 18. Nevertheless, cells implanted in bioinks frequently require gentle processing conditions and a soft matrix environment. Hence, bioink should be able to print with high shape fidelity and support cell function (the concept of the biofabrication window) 21. Only a few biomaterials fulfill such criteria and are suitable for bioprinting. In the past decade, many studies have provided strategies to improve dECM bioink printability. In this review, we introduce methods for producing dECM, summarize the strategies to improve dECM printability, and outline the applications, challenges, and prospects for dECM bioinks.

## Overview of dECM bioink

### ECM decellularization

**Figure 1:**
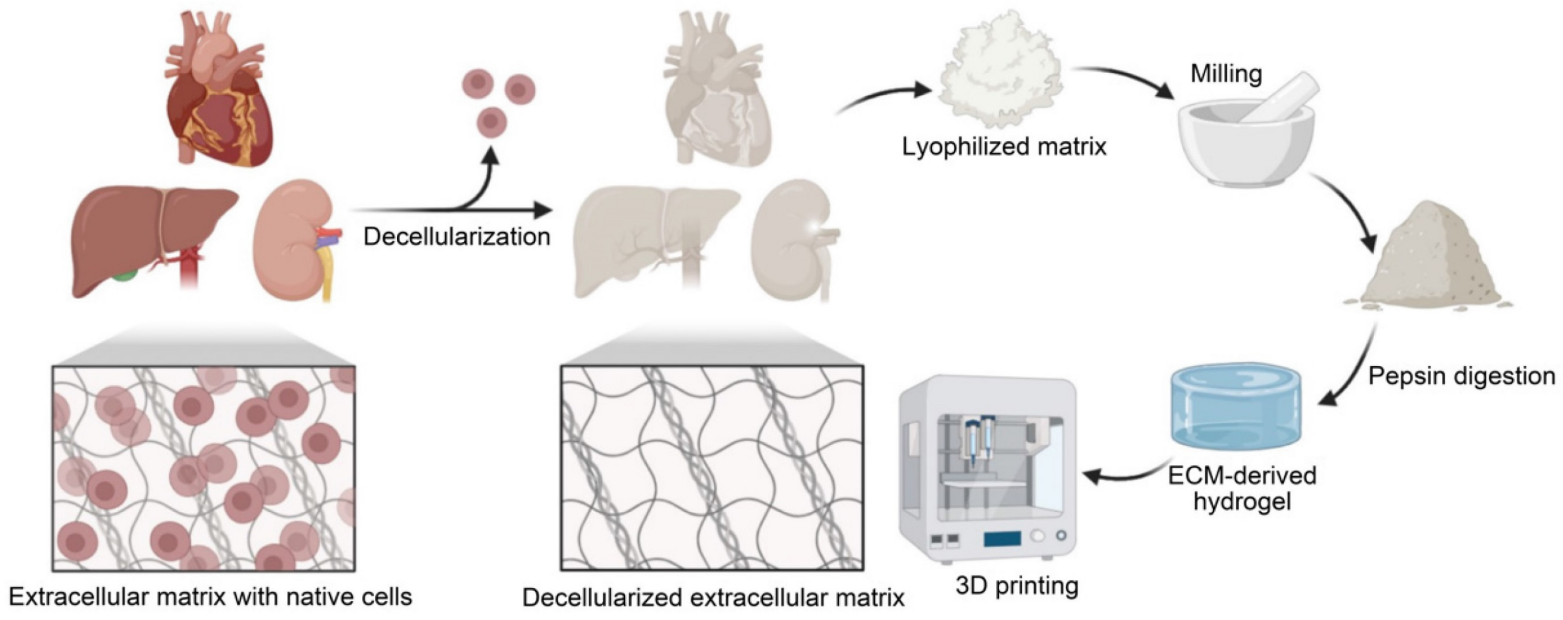
Fabrication of the dECM bioink. The tissue is first decellularized and lyophilized. After grinding, pepsin is then used to digest dECM. The pH and temperature can then be changed to induce gelation for 3D printing.

After removing any undesired tissue components, an appropriate decellularization process is required to remove cellular and immunogenic molecules from the tissues (**Figure 1**). During decellularization, it is essential to preserve the native ECM structure and components, such as GAGs, collagen, and growth factors 16. However, throughout this process, certain agents or procedures may negatively impact the composition or ultrastructure of the ECM. In general, gentler decellularization techniques preserve ECM components more effectively but may be less effective at eliminating unwanted cells. The opposite is true for more aggressive decellularization techniques. Thus, the choice of the decellularization process is crucial for deciding the final properties of the dECM bioink. Decellularization treatment methods are categorized as physical, chemical, enzymatic, and/or combinatorial 23. The tissue type, which determines tissue and cell density, ECM structure and composition, and fat content, are also important factors in choosing the decellularization procedure 12, 24.

#### Physical methods for decellularization

The freeze-thaw cycle method, high hydrostatic pressure, and supercritical CO2 extraction methods are common physical techniques for decellularization 25. By repeatedly freezing tissues at freezing temperatures and thawing them at ambient temperature or biological temperature, freeze-thaw cycles lyse cells. Rapid freezing frequently causes the intracellular formation of cytoplasmic crystals, membrane rupture, and cell death 12. Notably, this process typically requires numerous freeze-thaw cycles to attain optimal results 26, 27. Further, high hydrostatic pressure is used to kill tissue cells at pressures above 600 MPa 28, which is an efficient decellularization approach that preserves ECM ultrastructure 29. Lastly, supercritical CO2, a fluid created when the phase state of gases changes at a critical temperature and pressure, can remove biological components while preserving most of the GAGs and collagen content 30. Physical approaches are typically effective at protecting ECM structures but can be ineffective at removing cellular debris 31. Therefore, physical procedures must be followed by enzymatic or chemical treatments to eliminate cellular debris by facilitating decellularizing agent diffusion 32.

#### Chemical methods for decellularization

Detergents, acids, and alkaline chemicals are the most commonly used chemical agents in the decellularization process. Acidic and alkaline compounds effectively lyse cytoplasmic biomolecules and degrade nucleic acids 12. Various commonly used acid reagents are used for decellularization, including deoxycholic acid, hydrochloric acid, peracetic acid, sulfuric acid, and acetic acid 33. Further, sodium hydroxide, sodium sulfide, ammonium hydroxide, and calcium hydroxide are examples of alkaline reagents that are frequently employed 34. Various detergents can effectively dissolve cells by permeabilizing and solubilizing cell membranes 35. They comprise three main types: ionic, non-ionic, and zwitterionic. One of the advantages of the chemical-based decellularization approach is that it simultaneously sterilizes the final dECM by entering microorganisms and oxidizing microbial enzymes 36. However, chemical solutions can damage ECM components, particularly collagen triple helices at the fibrillar and molecular levels 37. Therefore, the decellularization procedure should be designed to utilize a combination of chemical treatments that will cause minimal damage to the ECM. In addition, the residual substances after decellularization must be considered, which can provoke a severe immunological reaction in the host. Maintaining biocompatibility, therefore, requires the complete elimination of the detergent.

#### Enzymatic methods for decellularization

The enzymes for decellularization, including proteases, nucleases, and esterases, remove the nucleic acid residues after cell rupture and cleave intercellular and extracellular junctions. Proteases, such as trypsin, cut the peptide bond between arginine and lysine 38. Nucleases, such as DNase and RNase, hydrolyze deoxyribonucleotide and ribonucleotide chains. Enzymes eliminate cellular and nuclear substances with remarkable selectivity. However, they are usually inefficient when applied alone and therefore need to be used in combination to carry out more thorough decellularization. Furthermore, these methods may dramatically reduce the GAG's content and degrade the ECM's ultrastructure 39. For example, prolonged exposure to enzyme agents may cause drastic damage to ECM ultrastructure and components, such as elastin, fibronectin, collagen, laminin, and GAGs 12, 24. In addition, the remaining enzymes in the dECM may cause significant adverse effects 40. Hence, enzymatic decellularization must be followed by thorough elution of the various residual biological and chemical substances to prevent an immunological response.

### Sterilization of the ECM

Sterilization, the final step of decellularization, eliminates harmful materials. Several sterilizing techniques exist, such as gamma-ray irradiation, electron beam irradiation, dry heat, pressured steaming, and the use of chemical agents (e.g., peracetic acid, ethanol solution, or ethylene oxide) 18. However, disinfection can affect the structural and mechanical properties of the final dECM. In addition to sterility, the preservation of structural stability and biocompatibility is important. Hence, selecting an appropriate sterilization method is imperative 41.

### dECM solubilization

After removing residual reagents, decellularized tissues can be lyophilized and milled to form a powder that can be dissolved in acidic environments via pepsin digestion. With physical stirring, it is possible to generate gel-like substances at a desired concentration 42. To deactivate pepsin, stop tissue digestion, and cause spontaneous reformation of intramolecular links in the solubilized dECM protein, the pH of the solution is neutralized to physiological conditions (pH = 7.4) 32, 43. One important advantage of using dECM bioinks for 3D printing is the ability of the pepsin-digested ECM solution to self-assemble into a cross-linked gel at physiological pH levels and temperatures via entropy-driven collagen kinetics 44. The dECM bioink is then prepared for 3DBP.

### Evaluation of the decellularization process

Residual cellular debris can be harmful and may generate inflammatory or immunological responses in hosts. The primary objective of decellularization is to remove as much cellular and genetic material from tissues as possible to eliminate the risk of immunogenicity 45. Moreover, damage to the ECM should be minimized throughout decellularization to preserve the native ECM components for TE. Therefore, to evaluate the effectiveness of decellularization, the content of the prepared dECM should be evaluated using qualitative and quantitative techniques. Commonly, hematoxylin and eosin (H&E) and 4′,6-diamidino-2-phenylindole (DAPI) staining are utilized to determine the efficacy of decellularization by staining cell nuclei and cytoplasm in decellularized tissues 16. Because of the immunogenicity of residual double-stranded DNA and the incomplete removal of cellular and nuclear material, the residual nucleic acid content should be determined 46. Therefore, the following three criteria were defined to indicate acceptable levels of leftover DNA after decellularization: 1) less than 50 ng of residual double-stranded DNA per mg of ECM dry weight; 2) DNA fragment lengths of < 200 bp; and 3) no visible nuclear material under H&E or DAPI staining 24, 47. Additionally, the content of ECM components, including GAGs, elastin, and collagen, should be evaluated.

## Strategies for improving dECM bioink printability

A variety of 3DBP techniques have been devised and applied to produce functional 3D structures, including extrusion-based printing techniques, digital light processing (DLP) techniques, inkjet-based printing techniques, fused deposition modeling technology, etc 4. These systems frequently produce 3D structures with a high level of fidelity through computer-aided design and manufacturing. In extrusion-based printing techniques, a bioink formulation is extruded through a nozzle to produce 3D structures. In droplet-based inkjet printing, low-viscosity cell suspensions are processed at high shear rates in the form of droplets. It is difficult to realize bioprinting with physiological cell density by inkjet printing technology at present, while low-viscosity materials will reduce the structural strength of printing molds, resulting in them not meeting the requirements of subsequent in vitro culture and transplantation. In addition, the process of inkjet printing may cause mechanical or thermal damage to cells. In DLP, UV light is projected onto a cell-laden polymer solution or prepolymer to spatially cross-link the solution. Extrusion-based bioprinting is the most commonly utilized bioprinting technology, as extrusion-based bioprinters are widely accessible, reasonably priced, and simple to operate. Fused deposition modeling technology is quite similar to extrusion printing, but additional heating changes the physical state of the material on its way through the printer head. This technology is widely used for thermoresponsive polymers and plastics. However, its application in bioprinting remains highly limited because of the high temperature needed to fuse the material and the fact that it is typically incompatible with cells 48. Due to the biological characteristics of dECM, printing methods such as FDM and inkjet-based printing are usually not used; extrusion-based printing or DLP bioprinting is mostly adopted.

Hydrogels can be extruded through nozzles to generate shape-stable gels, which are ideal for fostering cell proliferation in 3D environments. For extrusion-based 3DBP technology, bioinks are often required to have adequate rheological properties to keep the shape of extruded filaments and provide the desired printability. Printability is a nebulous topic that has been defined and quantified in recent years. The "excellent printability of a hydrogel" refers to a bioink's capacity to sustain pre-designed structures 49. Bioink printability can be semi-quantified based on the perimeter and area of square holes, which are calculated via ImageJ software using microscope images 50. Satisfactory printability largely depends on intrinsic features of the applied bioink, such as surface tension, mechanical characteristics, viscosity, and cross-linking mechanisms 21, 51. The mechanical characteristics of bioink can be measured by a specific value of Young's modulus or compression modulus. However, dECM gelation is relatively sluggish, potentially resulting in collapse due to gravity when printing hollow structures. This contributes to the poor 3D printability of dECM bioink and has substantially hindered the precision of multi-layer 3D structures 52, 53. To overcome the weak mechanical and low viscosity characteristics of dECM bioink, numerous strategies have been applied to enhance their printability (**Table 1**). Typically, these strategies can be categorized as physical or chemical. Physical strategies include support baths, sacrificial polymers, and leveraging an external supporting structure, such as polycaprolactone (PCL) 54, 55 or poly (ethylene/vinyl acetate) (PEVA) 56, 57, to preserve the dECM solution during gelation when printed. The chemical approach aims to refine dECM-based bioink designs to improve storage modulus and yield stress to prevent the buckling of suspended filaments and minimize construction deformations. This includes combining dECM-based bioinks with other quick cross-linking systems [e.g., dECM/alginate, dECM, and gelatin methacrylate (GelMA)] 58. Cross-linking agents and methacryloyl functional groups can be added to chemically modify dECM bioinks to improve their printability 59-62.

The polymers adopted to improve dECM printability are both natural and synthetic; these are the most frequently used biomaterials for 3DBP 63. Many natural polymers originate in the natural ECM or are extracted from marine organisms. Collagen, gelatin, alginate, fibrin, and chitosan, for example, have been widely utilized in bioprinting 64. Its superior degradability, low immunogenicity, and biocompatibility make it appropriate for bioprinting 65. Moreover, natural polymers can be chemically changed to improve their characteristics or to induce further cross-linking (e.g., thiolated hyaluronic acid and GelMA). Typically, these modifications increase the material's physical properties, such as higher and stronger reticulation, resulting in slower degradation and increased durability. Nonetheless, natural polymers have drawbacks. They lack the repeatability of synthetic polymers and are more challenging to functionalize than synthetic polymers. Synthetic polymers can be classified as non-biodegradable or biodegradable. For engineering bones and cartilage, nonbiodegradable synthetic polymers Polyethylene glycol (PEG) is the most widely utilized. Biodegradable synthetic polymers, such as poly (lactic acid) and PCL, can degrade at a specific rate under natural conditions 66. Synthetic polymers are distinguished by their controllable mechanical qualities and structural stability 67. Synthetic polymer-based bioinks are largely bioinert and display poor cell viability compared to natural biopolymer-based bioinks. But they can function as supports and intercalated layers to improve the mechanical properties of the final 3D construction. The role of these polymers in increasing the printability of dECM is summarized in **Table 1**.

### Physical strategies for improving dECM bioink printability

The effects of gravity and time-dependent flow prior to cross-linking on printed filaments can be mitigated by providing support to the bioink or printing in a buoyant environment. As with conventional 3D printing, supportive polymeric materials such as PCL 54, 55 or PEVA 56, 57 can provide the initial geometrical restriction to extruded ECM hydrogel precursor solutions 17, 22. Likewise, sacrificial materials and support baths based on thermosensitive hydrogels (e.g., gelatin 68, 69 or poloxamers 70, 71) can also be used to print temporary supports 72. For example, the freeform reversible embedding of suspended hydrogels (FRESH) 73 allows the 3D geometry of the printed soft materials to be retained in a support bath. The printed scaffold can be acquired by melting the support bath after solidification.

**Figure 2:**
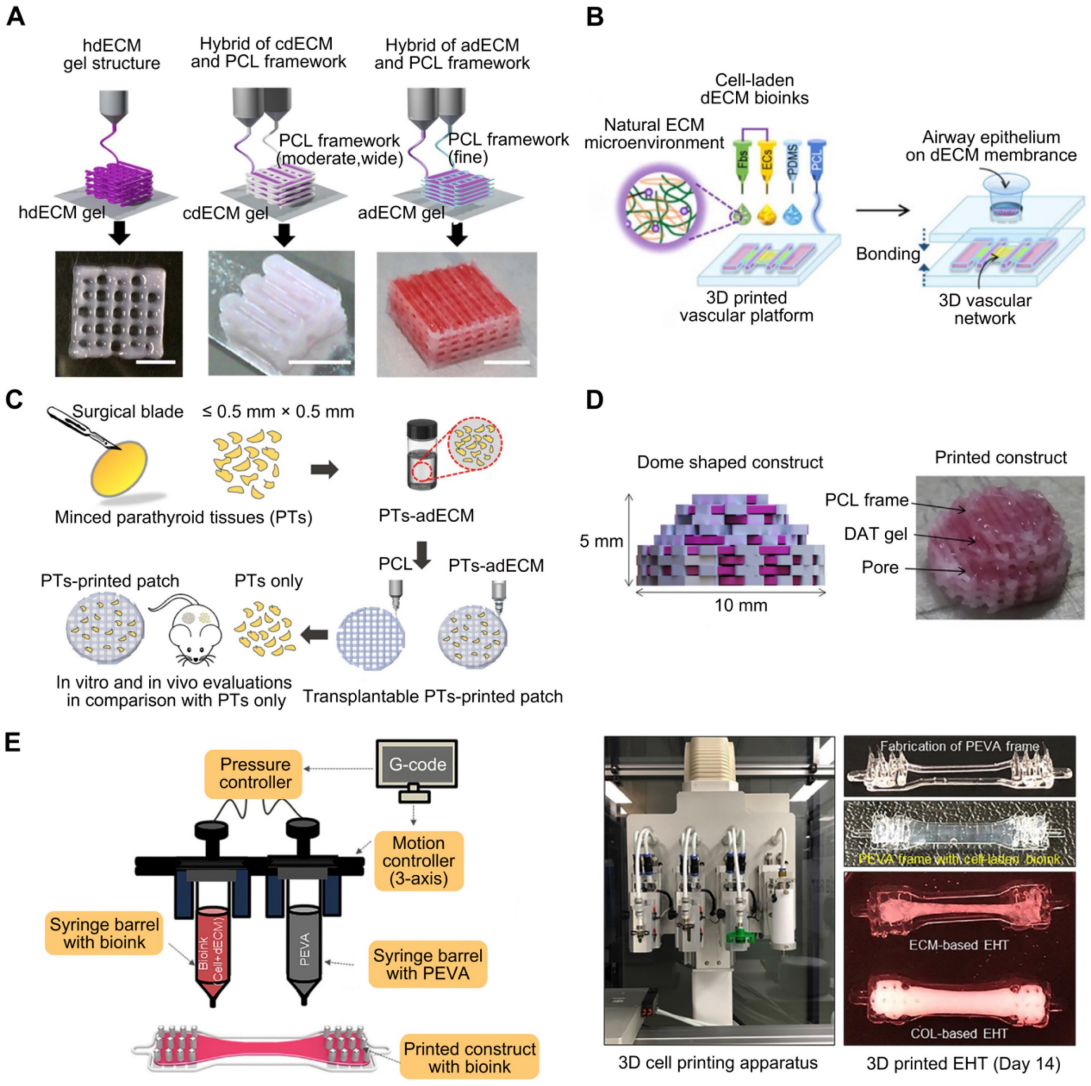
Polymeric polymers that provide structural support for dECM-based bioink. (A) With the PCL framework, heart dECM (hdECM), cartilage dECM (cdECM), and adipose dECM (adECM) were printed (adapted with permission from 22, copyright 2014 Springer). (B) dECM bioink derived from pig tracheal mucosa was printed on a PCL frame support (adapted with permission from 77, copyright 2018 IOP Publishing). (C) Adipose-derived dECM (adECM) bioink encapsulated with parathyroid glands was printed on a PCL mesh (adapted with permission from 78, copyright 2021 IOP Publishing). (D) The PCL framework provides the framework for breast reconstruction (adapted with permission from 55, copyright 2015 Elsevier). (E) Heart-derived dECM-based bioink was extruded into a PEVA frame to create an engineered heart tissue model (adapted with permission from 56, copyright 2019 Elsevier).

PCL is biocompatible, biodegradable, and possesses physicochemical and mechanical qualities such as viscoelasticity and formability 74. Although PCL, being water insoluble, cannot be used to encapsulate cells 75, constructs can be printed in combination with layers of PCL to ensure the scaffold's long-term rigidity, even if the cell-encapsulating material degrades rapidly 76. Pati et al. 22 used cartilage, heart, and adipose-derived dECM bioinks to manufacture tissue constructions, in which PCL was the framework used to maintain the shape of the printed structures (**Figure 2A**). With the bioprinted designs, they achieved good cell survival rates, cell line-specific gene expression, and ECM production. Similarly, dECM bioink produced from swine tracheal mucosa was successfully printed with a PCL frame support to build a functioning in vitro airway-on-a-chip connected to a vascular network 77. These bioprinted chips exhibited respiratory symptoms (i.e., asthmatic airway inflammation and allergen-induced asthma aggravation) in a physiological environment (**Figure 2B**). Yu et al. 78 printed adipose-derived dECM bioink encapsulated with parathyroid glands onto a PCL mesh (**Figure 2C**). In vitro and in vivo observations indicated that parathyroid-printed patches could reverse surgery-induced lifelong hypoparathyroidism. For breast reconstruction, a recent study designed a dome-shaped 3D cell-printed construct made of decellularized adipose tissue matrix bioink and a PCL framework that prevented structural collapse (**Figure 2D**). The results demonstrated that the constructs promoted host cell infiltration and adipose tissue formation 55. This confirmed that PCL effectively served as a framework for printing dECM and enhanced its geometrical precision and mechanical durability 79. However, PCL is a hydrophobic and physiologically inactive polymer, which can hamper cell adhesion and increase the structural heterogeneity of the scaffolds. The interaction between biomaterial surfaces and cells is critical. Surface wettability is a crucial biomaterial characteristic that helps modulate protein adsorption and cell behavior 80.

PEVA, a non-biodegradable thermoplastic derived from ethylene and vinyl acetate, has an elastic modulus between 10.22 and 13.86 MPa, making it suitable for providing mechanical support to bioinks 81, 82. For example, Das et al. 56 extruded pepsin-digested, heart-derived dECM-based bioink into a PEVA frame to fabricate a heart tissue model (**Figure 2E**). In this model, needle-like PEVA posts helped generate mechanical strain in encapsulated cardiomyocytes, which may then influence cardiomyocyte alignment. The dECM-based structures enhanced cardiomyocyte development and differentiation.

**Figure 3:**
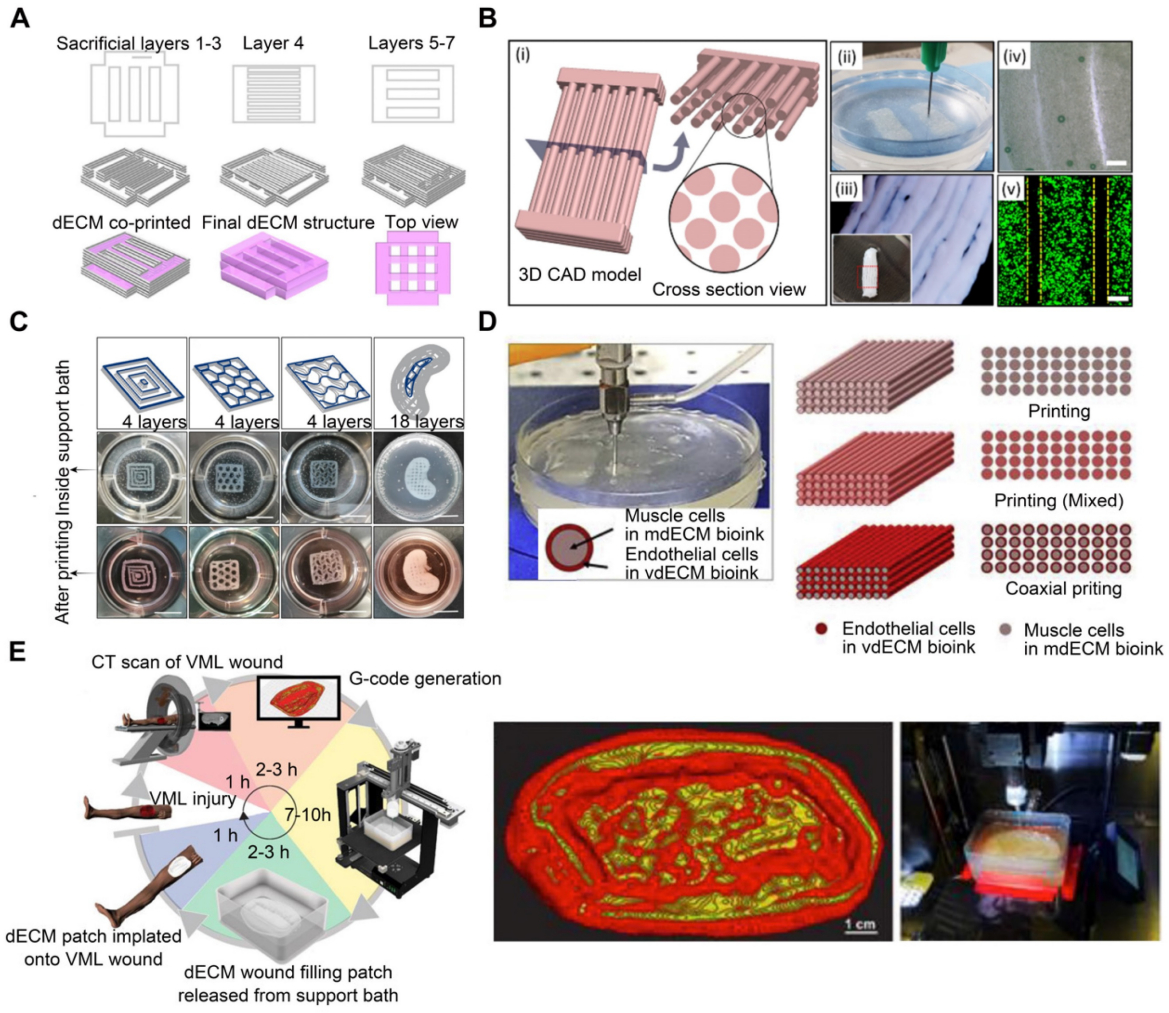
Support baths are used for fabricating dECM-based structures. (A) Pluronic F-127 support sacrificed structure for supporting the dECM bioink (adapted with permission from 85, copyright 2019 Elsevier). (B) A multilayered tendon/ligament constructs printing by using a gelatin granule-supporting bath (adapted with permission from 88, copyright 2022 IOP Publishing). (C) Images of the constructs inside the agarose support bath and after removal of the agarose support bath (adapted with permission from 92, copyright 2021 IOP Publishing). (D) Muscle-derived bioink encapsulated with human skeletal muscle cells was printed in a gelatin granule-based printing reservoir to reconstruct skeletal muscle (adapted with permission from 95, copyright 2019 Elsevier). (E) dECM-based bioink was used to create the ECM hydrogel patch in a FRESH support bath (adapted with permission from 73, copyright 2022 Wiley).

Sacrificial materials have been extensively studied to improve geometrical characteristics via the molding of non-sacrificial materials around sacrificial components, which are subsequently removed from the sacrificial components. Sacrificial materials can provide the mechanical conditions for dECM bioprinting without using permanent polymer composites 83. For instance, Pluronic F-127, a commonly used sacrificial 3D-printable material, exhibits reversible gelation and melting at room temperature (23 °C) and refrigeration temperature (4 °C) 84. Additionally, the geometry of the printed structures can be adjusted based on the Pluronic F-127 printing parameters. To generate stable structures and regulate the orientation and geometry of in vitro-grown biliary trees, Lewis et al. 85 printed a Pluronic F-127 sacrificial support structure and extruded the dECM bioink into it. Irreversible gelation of dECM hydrogels enabled the removal of the Pluronic F-127 sacrifice so that only the dECM hydrogel 3D structure remained (**Figure 3A**). In conclusion, the use of sacrificial methods to construct vascular networks has the potential to create complicated vascular systems in 3D TE constructs. However, the total removal of the sacrificial material is not always convenient, and any residual materials may be cytotoxic to the host 86.

Using reversible support baths to achieve a 3DBP construct is effective for most low-viscosity materials; moreover, it enables the printing of complex structures without the need for additional support structures 87. Chae et al. utilized a gelatin granule-based printing technique to construct freestanding multilayered dECM-based tendon/ligament structures (**Figure 3B**) 88. The escaped bath components were removed during the cross-linking of the printed constructs at 37 °C, owing to the thermally reversible characteristics of gelatin. The resultant constructs displayed a porous, structured architecture with longitudinally aligned patterns 89. Enzymes, such as transglutaminase, sortase, lysyl oxidase, tyrosinase, phosphatase, and peroxidase, are also utilized in alternative cross-linking techniques for natural and manufactured hydrogels 90, 91. For enzymatic cross-linking of dECM, Sobreiro-Almeida et al. utilized an agarose microparticle support bath impregnated with microbial transglutaminase (**Figure 3C**) 92. The bath was capable of self-healing, following nozzle movement and bioink deposition 93, 94. Further, its usage improved the mechanical properties of the bioink and the integrity of the bioprinted structure. This approach is particularly effective for obtaining structures with good print resolution and structural integrity. In another study, muscle-derived bioink encapsulated with human skeletal muscle (SM) cells was printed in a gelatin granule-based printing reservoir to address the issue of mechanical stiffness and low viscosity of the bioink (**Figure 3D**) 95. The addition of polyvinyl alcohol (PVA) coagent to the gelatin granules allowed the dECM bioink to rapidly polymerize without loss of structural fidelity after extrusion into the granule-based reservoir system. This structure was later used in a model of volumetric muscle loss (VML), mimicking its hierarchical architecture of vascularized muscle. Notably, when muscle-derived bioink was combined with vascular tissue-derived bioinks containing human umbilical vein endothelial cells (HUVECs) and printed through a coaxial nozzle, functional recovery was enhanced by up to 85% compared to that in uninjured tissues. These findings demonstrated the application of 3DBP using dECM bioinks to replicate the SM hierarchical architecture. In addition, Feinberg et al. 96 created the FRESH technique for extruding hydrogel bioinks into a second hydrogel that served as a support medium. During printing, they utilized a bath of gelatin microparticles that behaved like Bingham plastic-rigid bodies and viscous fluids at low and high shear stresses, respectively. Thus, a needle-shaped nozzle encountered minimal mechanical resistance as it passed through the bath, but hydrogels that extruded from the nozzle and were deposited within the bath were retained in situ. In this support bath, the intended 3D geometry of printed soft materials was maintained. The scaffold was easily removed after solidification by melting the support bath. One study 73 adopted the FRESH 3DBP method to print a high-concentration bioink consisting of dECM and type I collagen based on computerized tomography imaging data to produce patient-specific dECM patches for implantation into canine VML wound models (**Figure 3E**). The FRESH gelatin microparticle support bath introduced microporosity to the scaffolds and aligned the dECM hydrogel to the wound geometry.

### Chemical strategies for improving dECM bioink printability

To eliminate the necessity of supporting materials when generating mechanically stable structures using dECM-based bioinks, the printability of dECM bioinks can be enhanced by combining cross-linkable hydrogels 97, 98. The dECM supplies tissue-specific biochemical components, while the cross-linkable hydrogels improve the mechanical qualities of the printed constructs. Even though cross-linkable hydrogels and dECM-based bioinks can be combined to enhance their mechanical properties, the dECM bioinks can also be directly cross-linked via methacrylation 61, 62. A common technique to stabilize extruded filaments is photo-induced cross-linking. For photo-cross-linking, photo-initiators activate free radical reactions under ultraviolet (UV) light irradiation. Upon exposure to light, photo-initiator molecules added to the bioink formulation generate reactive matter that initiates polymerization. The commonly used photo-initiators and photosensitizers include 1-[4-(2-hydroxyethoxy)phenyl]-2-hydroxy-2-methyl-1-propan-1-one 99, 100, lithium phenyl-2,4,6-trimethylbenzoylphosphinate 100, 101, and ruthenium/sodium persulfate (dERS) 102. Each photo-initiator requires a specific wavelength of light for cross-linking; the optimal wavelength for lithium phenyl (2,4,6-trimethylbenzoyl) phosphinate is 405 nm, and some wavelengths may damage cells. Recently, a white light technique called the Eosin Y system was devised to swiftly induce cross-linking without causing cell damage 103. In extrusion printing, photo-cross-linking can be performed either after each layer has been deposited or after the entire print has been completed, with the latter requiring superior ink shape preservation. However, preventing filament collapse remains a challenge in both cases 104, 105.

**Figure 4:**
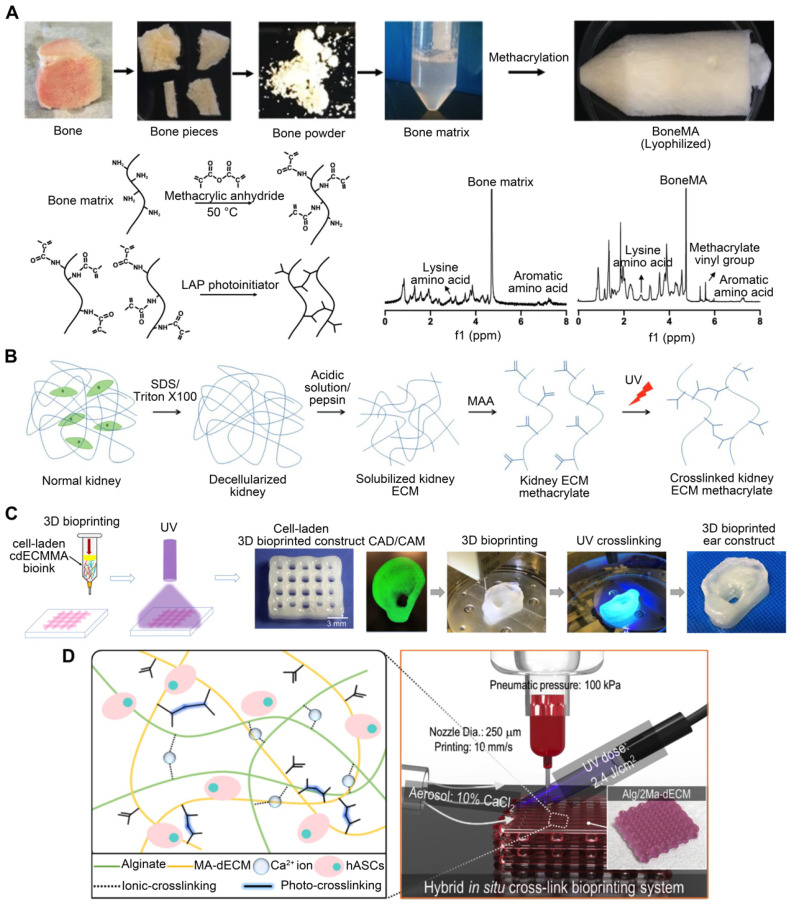
dECM bioink printability is improved by functionalizing with methacrylate groups. (A) The bone matrix is subjected to an addition reaction with methacrylic anhydride in which methacrylate groups are connected to the pendant amine groups (adapted with permission from 109, copyright 2021 IOP Publishing). (B) Illustration of kidney-specific photo-crosslinkable ECM hydrogel production (adapted with permission from 61, copyright 2019 Wiley). (C) 3D bioprinting technique employing photo-crosslinkable cartilage-derived ECM bioink for auricular cartilage regeneration for customized auricular reconstruction (adapted with permission from 110, copyright 2021 Elsevier). (D) The ECM derived from cartilage was methacrylated into a photo-crosslinkable hydrogel and combined with chondrocytes to create a printable bioink (adapted with permission from 111, copyright 2020 Elsevier).

Depending on their origin, native tissues exhibit a wide range of mechanical properties in nature 106. The osteogenic potential of bone dECM has been researched 107. However, bone dECM hydrogels have a lower G' value (~150 Pa at 6 mg/mL) than natural bone (8-11 GPa) 108. For applications such as bone tissue, which require much higher moduli, dECM bioink must enhance its elastic modulus and printability. For example, bone-derived dECM bioink can be functionalized with methacrylate groups (**Figure 4A**) 109. Methacrylation of bone-derived biomaterial allows photo-cross-linking in the presence of a photo-initiator while maintaining the biological benefits of the native ECM composition. The mechanical properties of the biomaterial can be changed, with the elastic modulus increasing as a function of the photo-cross-linking duration. Ali et al. 61 successfully conferred photo-cross-linking characteristics via methacrylation to kidney dECM to print functional kidney microtissues in vitro without necessitating additional polymeric materials (**Figure 4B**). Importantly, this kidney-specific dECM-based bioink formulation supported the maturation and tissue development of human kidney cells in a kidney-specific microenvironment. Visscher et al. 110 developed a photo-cross-linkable cartilage-derived ECM bioink for auricular cartilage regeneration (**Figure 4C**). The cartilage-derived ECM was methacrylated into a photo-cross-linkable hydrogel and combined with chondrocytes to create a printable bioink. After methacrylation, the bioinks exhibited adequate mechanical properties, with a stiffness of 25050 ± 2573 Pa, and were printed into an anatomical ear shape. Lee et al. 111 developed an alginate/methacrylated-decellularized bone ECM bioink to fabricate 3D cell-laden mesh structures for bone TE (**Figure 4D**). Compared to pure alginate bioinks, composite architectures can significantly increase cellular activity. Kim et al. 62 used dECM methacrylate derived from porcine SM, combined with sacrificial fibrillated PVA, to fabricate a uniaxially aligned/micro-topographical SM structure. Using this dECM-based material and extrusion bioprinting technology, fibrillated PVA was aligned using regulated wall shear stress within a micronozzle as a sacrificial material. UV light was used to cross-link the printed structures, and PVA components were removed during structure stabilization. This innovative technology can produce uniaxially aligned fibrillated printed constructs capable of inducing myoblast orientation and, thus, accelerating myogenic development. Owing to the combined influence of SM-specific biochemical and topographical cues, the myoblasts present in the 3D-printed structure aligned and differentiated to a high degree, resulting in a high level of myotube formation. To create biological auricle substitutes with exact geometries and low immunogenicity, Jia et al. 112 utilized auricular chondrocytes and a bioactive bioink consisting of a biomimetic microporous methacrylate-modified decellularized cartilage matrix, in addition to GelMA, poly (ethylene oxide), and PCL. Using 3DBP technology, it was possible to precisely control the distribution of chondrocyte-laden bioinks and PCL to create auricular substitutes with the desired form and dynamics. Finally, mature auricular cartilage tissue with good elasticity, many cartilage lacunae, and cartilage-specific ECM deposition were observed in nude mice.

**Figure 5:**
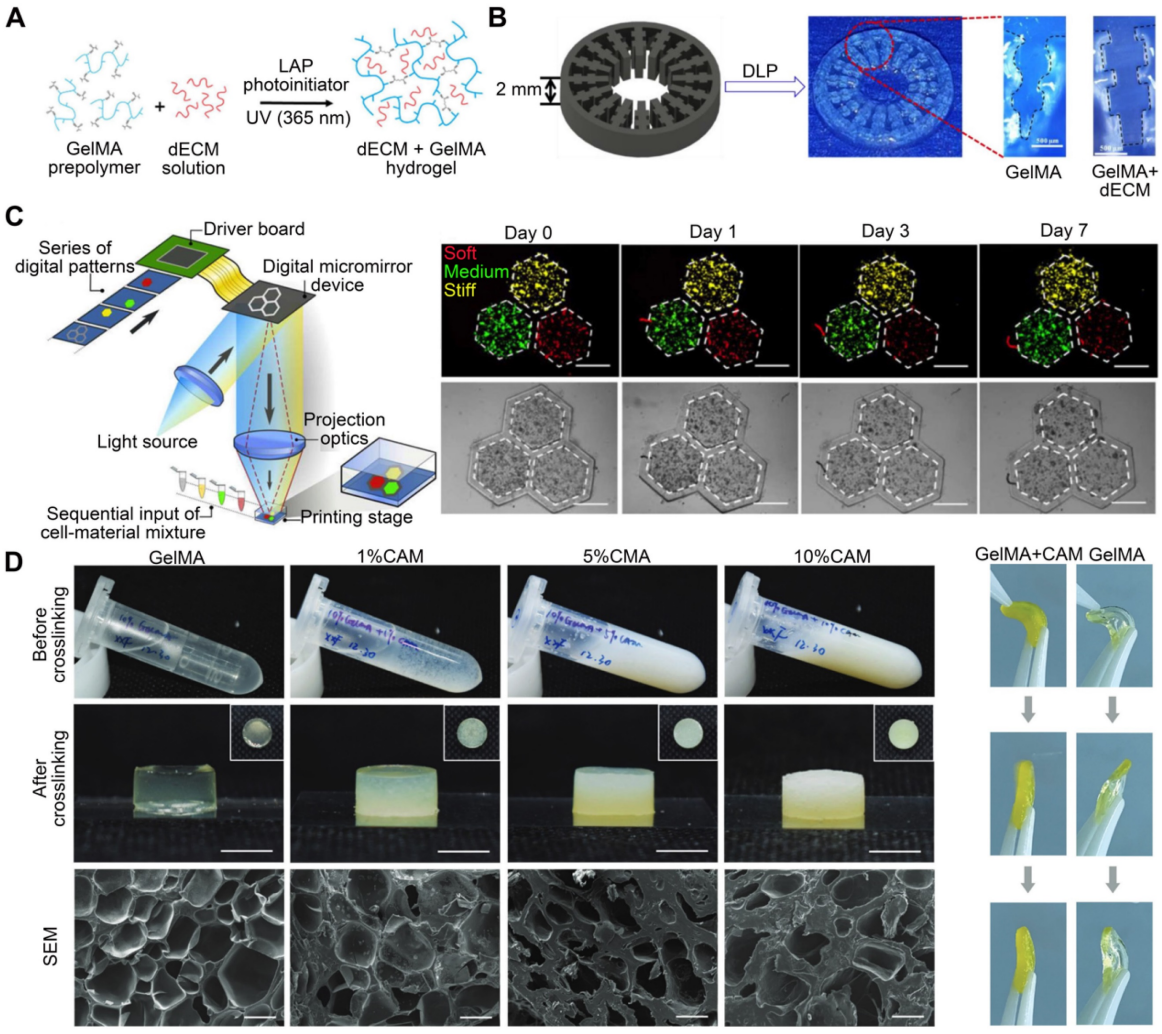
dECM bioink printability is improved through the incorporation of GelMA. (A) Cross-linking mechanism of dECM-based hydrogel containing GelMA (adapted with permission from 115, copyright 2019 Elsevier). (B) dECM derived from pig liver tissues mixed with GelMA was used to build a liver microtissue structure resembling an inner gear (adapted with permission from 98, copyright 2020 Wiley). (C) dECM-based bioink with different stiffness values was used for printing to mimic a cirrhotic liver environment and to bioprint a liver stiffness model (adapted with permission from 97, copyright 2018 Elsevier). (D) Bioinks containing auricular tissue-derived dECM of different concentrations and GelMA are used to print auricular structures (adapted with permission from 116, copyright 2022 Wiley).

GelMA, a gelatin with a photopolymerizable methacrylamide group, is widely used 113. Adjusting its concentration and printing parameters, such as UV light exposure time, printing temperature, and photo-initiator quantity, can modify the elastic modulus of methacrylate based on the degree of methacrylation 114. Yu et al. 115 utilized a GelMA and dECM bioink formulation for DLP-based printing to improve the mechanical properties of heart-derived dECM bioinks to print heart structures (**Figure 5A**). When exposed to UV light at different times, the compression modulus of the hydrogel ranges from approximately 1.5 to 6.5 kPa. This dECM-based biomaterial for in vitro disease modeling featured biomimetic architecture and modifiable mechanical properties. For example, different bioink stiffness values were used for printing to mimic a cirrhotic liver environment and evaluate cellular behavior. When exposed to UV light at times of 10 s, 20 s, and 40 s, the compression modulus values of the hydrogel are roughly 0.5 kPa, 5 kPa, and 15 kPa, which each correspond to the softer than healthy range (soft), healthy liver range (medium), and cirrhotic range (stiff), respectively. HepG2 cells encapsulated in a stiff cirrhotic-like substance exhibited decreased proliferation and elevated invasion markers (**Figure 5C**) 97. Mao et al. 98 obtained dECM by decellularizing porcine liver tissues and generating GelMA/dECM cell-laden bioink to create a gear-like liver microtissue structure via DLP-based 3DBP (**Figure 5B**). In vitro experiments showed that dECM significantly enhanced HepG2 cell activity and proliferation, liver function, and metabolism. In addition, the print resolution of DLP 3DBP technology was greater than that of extrusion printing. Xie et al. 116 mixed auricular tissue-derived dECM with GelMA solution for DLP bioprinting (**Figure 5D**). The blended bioink possessed the requisite mechanical properties, swelling ratio, and printability and could produce auricular structures with high elasticity and high printing precision. Based on a multilayer biomimetic strategy, Jian et al. 117 integrated GelMA and meniscal ECM to simultaneously evaluate printability and cytocompatibility. They utilized 3DBP technology to combine the benefits of PCL and meniscal fibrocartilage chondrocyte-loaded GelMA/meniscal ECM bioinks. This produced a biomimetic scaffold whose mechanics, components, and microenvironment resembled the native meniscus. Although these studies demonstrated the advantages of stereolithography, such as its intrinsic high resolution and the ease of fabricating complex scaffolds 118, 119, stereolithography can only process solutions containing UV-activated photo-initiators. However, exposure to UV light can reduce cell viability, especially when cross-linking multilayer structures. In addition, photoinitiators are generally cytotoxic. To ensure high cell viability, it is necessary to carefully examine the photo-initiator concentration and UV exposure to design a cell-preserving polymerization process 120, 121.

Hyaluronic acid methacrylate (HAMA), derived from the methacryloylation of hyaluronic acid, can rapidly undergo gelation with lithium phenyl (2,4,6-trimethylbenzoyl) phosphinate under UV irradiation 122. HAMA has good biocompatibility and degrades in the presence of hyaluronidase 123. Wang et al. 124 created a unique tissue-specific bioink by mixing pancreatic ECM with HAMA. When the dECM concentration was 10 mg/mL and 20 mg/mL, the Young's modulus of the hydrogel was 8.3 ± 0.3 kPa and 7.1 ± 0.3 kPa, respectively. The HAMA/ECM hydrogel maintained islet cell adhesion and morphology via the Rac1/ROCK/MLCK signaling pathway in vitro, hence enhancing islet cell function and activity. Kim et al. 125 prepared a novel bioink using dECM microparticles instead of the conventional solubilized form. They manufactured 3D liver structures by adding dECM powders to a gelatin slurry containing hyaluronic acid and fibrinogen. The compressive modulus of the cartilage dECM hydrogels was approximately 280 Pa, which was significantly less than the mechanical requirements of the meniscus 126. To produce mechanically resilient, multilayer scaffolds, Barthold et al. 127 developed a biomaterial ink containing ECM particles. Using the sulfhydryl groups on the cysteines of decellularized articular cartilage for cross-linking with thiol-functionalized hyaluronic acid, disulfide connections were formed between the two biomaterial ink components to form a 3D network. After the hydrogel forms a network, the Young's modulus increases quickly to 300 kPa, approaching the level of native cartilage (500 kPa-1 MPa).

**Figure 6:**
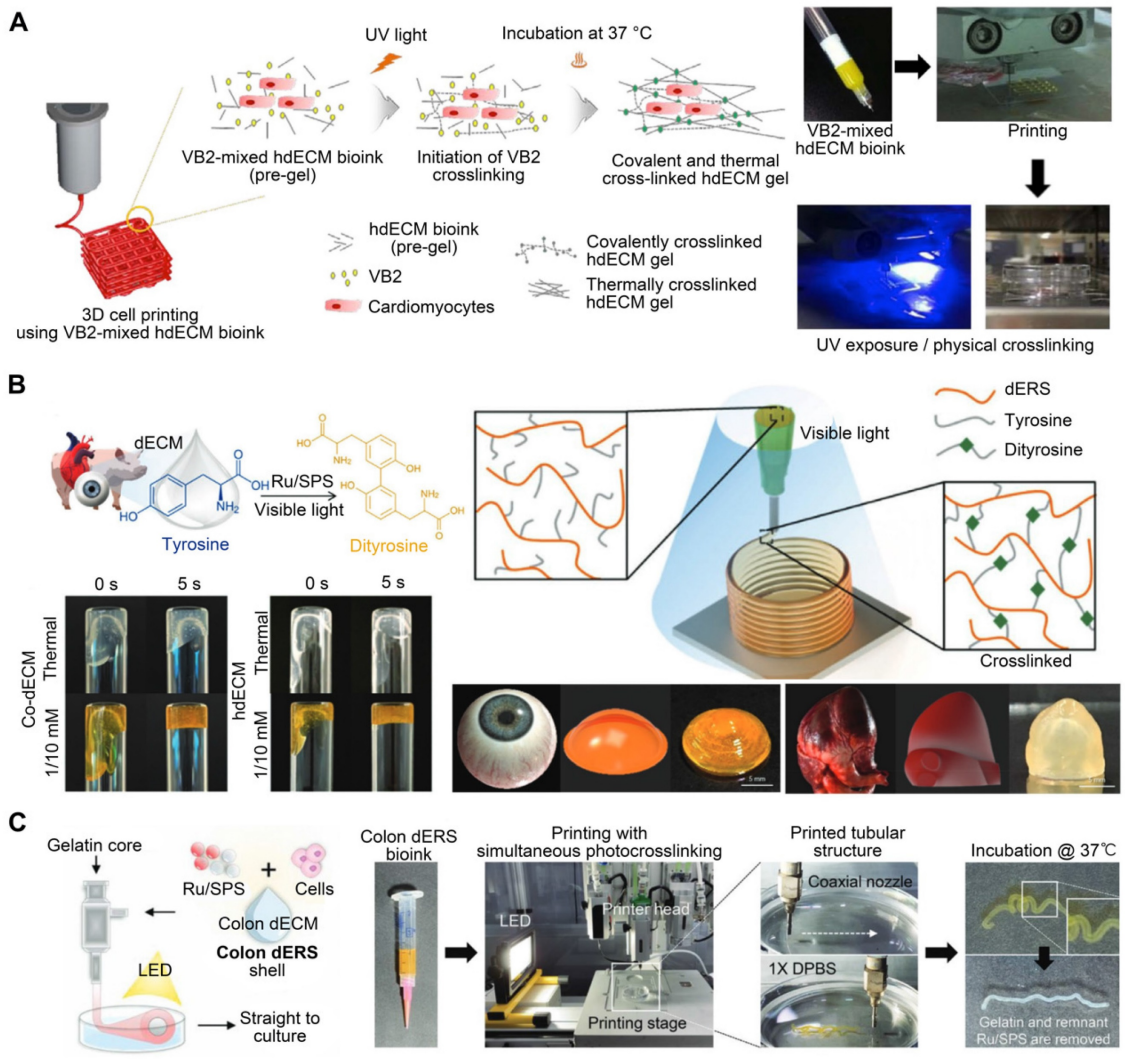
The photo-crosslinking agent is added to improve dECM bioink printability. (A) A schematic illustration of the two-step crosslinking mechanism that involves vitamin B2, thermal crosslinking, and concurrent covalent crosslinking (adapted with permission from 18, copyright 2016 Elsevier). (B) A light-activated cross-linking reaction involving tyrosine in dECM-based bioink and the use of dERS with a light-activated cross-linking system for centimeter-scale 3D printing of high-aspect-ratio structures (adapted with permission from 102, copyright 2016 Wiley). (C) A tubular intestine model was established using dECM derived from the colon and supplemented with a dERS photoinitiator (adapted with permission from 133, copyright 2022 Wiley).

Vitamin B2 is a biocompatible photo-crosslinking agent 128. To manufacture cardiac constructions with enhanced mechanical properties, Jang et al. 18 added Vitamin B2 as a photo-initiator to a solution of pepsin-digested dECM. Each printed layer was subjected to UV light to initiate photo-crosslinking (**Figure 6A**). The printed heart construct had up to 10 layers, with mechanical stiffness comparable to that of native cardiac tissue. The dERS were cross-linked via an oxidation mechanism driven by visible light. This pathway is triggered by visible light (400-450 nm), inducing the formation of tyrosyl free radicals that form covalent dityrosine cross-links with adjacent tyrosine molecules. Tyrosine is a common amino acid that controls structural conformation changes in proteins 129. Kim et al. 102 developed a novel light-activated dECM bioink using dERS. Other dECM hydrogels have compressive moduli that range from 0.18 to 3.0 kPa, according to previous studies 18. However, the dERS product revealed a significantly enhanced compressive modulus value of up to 86.4 kPa. The materials were polymerized using a dityrosine-based crosslinking technique with fast reaction kinetics and enhanced mechanical characteristics (**Figure 6B**). Ru2+ undergoes photolysis in the presence of visible light and an electron acceptor to produce Ru3+, which oxidizes aromatic residues like tyrosine. The oxidized tyrosine groups are converted into tyrosyl radicals, which are then neutralized by the formation of covalent dityrosine bonds 130. Moreover, owing to the high visible light absorption of Ru and its chemical stability in an excited state, the dERS cross-linking method was evidenced to be rapid and highly effective 131. Collagen, which contains tyrosine, is the most abundant protein in the ECM 132. Given that tyrosine is plentiful in dECM bioinks, the dERS system can facilitate the rapid cross-linking of dECM bioinks to generate complicated constructs with high aspect ratios. Similarly, a colon-derived dECM supplemented with a dERS photo-initiator was developed 133 and used to establish a tubular intestinal model. During coaxial printing, the photo-initiator dERS improved the shape accuracy of tubular structures (**Figure 6C**). Validation analyses of bioprinted tubular structures as prospective intestinal models demonstrated the successful creation of intestinal tissues with high levels of enteroendocrine marker expression 133.

Polyethylene glycol diacrylate (PEGDA) is a common synthetic polymer used in the production of hydrogel biomaterials with flexible elasticity, solute permeability, and biocompatibility. PEGDA enabled rapid photopolymerization after printing 134. Adding PEGDA to solubilized dECM improved bioink viscosity and enabled greater control over the mechanical properties of tissues 17. The bioink could be cross-linked via two processes to adjust its structural rigidity. The two-step mechanism for cross-linking involved spontaneous crosslinking of thiol groups with PEG acrylate groups and UV photopolymerization of thiol and PEG alkyne groups. Through these processes, the Young's modulus values of the bioink range from approximately 100 Pa to 20 kPa. Zhu et al. compounded cartilage dECM with PEGDA and honokiol (Hon, a natural anti-inflammatory chemical) to construct cartilage scaffolds using 3D printing technology. After LPS treatment, the levels of pro-inflammatory factors TNF, IL-1, and IL-6 released from macrophages co-cultured with the PEGDA/ECM scaffolds increased considerably. However, adding Hon inhibited the release of these pro-inflammatory factors. In addition, in vitro animal tests demonstrated that the PEGDA/ECM/Hon scaffold stimulated cartilage and bone tissue regeneration in osteochondral lesions 135. In another study, Shin et al. 136 coupled dECM-based bioink with Laponite nanoclay and PEGDA to build structures with tunable mechanical characteristics. The compressive modulus of the bioinks was tunable from 13.4 to 89 kPa by varying the amount of PEGDA in the bioink formulation. This naturally derived bioink could be used for unsupported printing as it cures quickly and promotes the high viability of encapsulated cells. Laponite ensured flawless extrusion during the production process and maintained precision during the stacking procedure, while PEGDA enabled rapid photopolymerization after printing 137. In addition, altering the concentration of PEGDA allowed for more precise control over the ultimate rigidity of the printed structures. However, while the suitable addition of PEGDA enhanced the cross-linking network, the excessive addition of PEGDA (because it is a short-chain molecule) could diminish the gel's toughness. Moreover, the degradation byproducts of composite polymers, such as PEGDA and cross-linkers, may be cytotoxic, resulting in increased inflammation 138. In these cases, chemical methods may unavoidably reduce the native bioactivity of pristine dECM because its physiological components are affected, contradicting the original purpose of using dECM bioinks to imitate native tissue microenvironments.

**Figure 7:**
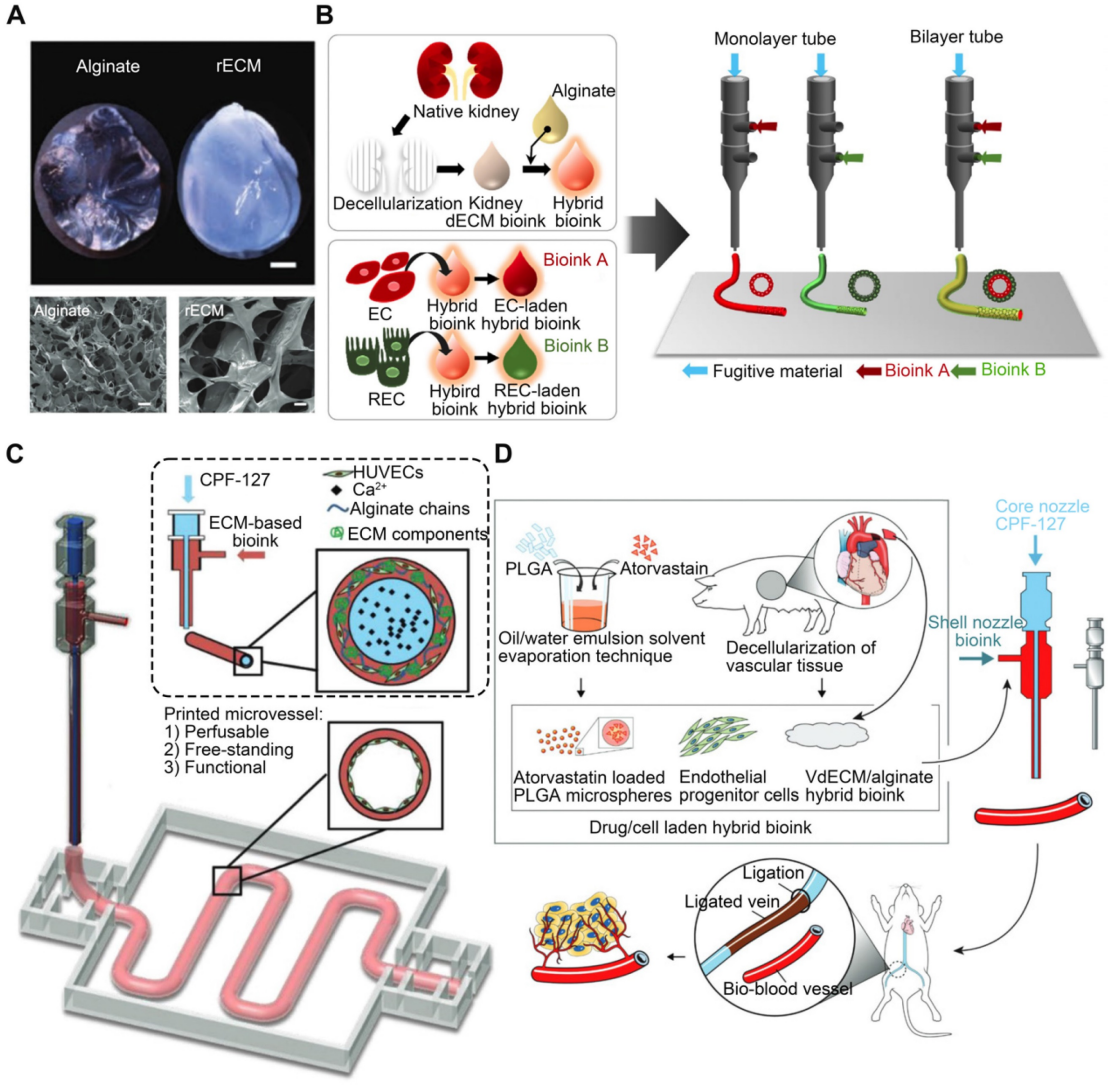
Alginate is added to improve dECM bioink printability. (A) Image of mouse ECM and alginate hydrogels (adapted with permission from 140, copyright 2021 Elsevier). (B) Alginate and kidney-derived dECM are combined to recreate the native renal microenvironment (adapted with permission from 58, copyright 2020 Elsevier). (C) Coaxially cell-printed vessels using VdECM/alginate hybrid bioink containing HUVECs (adapted with permission from 59, copyright 2018 Wiley). (D) Mixing VdECM and sodium alginate produced a hybrid bioink that was used to encapsulate atorvastatin/poly(lactic-co-glycolic acid) microspheres and endothelial progenitor cells (adapted with permission from 145, copyright 2017 Wiley).

Alginate, a bioinert and biocompatible material, undergoes rapid cross-linking and gelation when exposed to calcium ions. Thus, this method is commonly employed to produce double-network hydrogels 139. Alginate can improve dECM mechanics, as evidenced by De Santis et al.'s research, in which they reinforced dECM bioinks with alginate (**Figure 7A**) 140. Alginate permits quick gelation following ionic cross-linking while maintaining the phase-separated ECM in cross-linked hydrogels, achieved via micro-scale phase separation as opposed to macro-scale phase separation 141. By adjusting the ratio of alginate to dECM, it is possible to preserve biological functions at various stages of tissue development, including tissue-specific differentiation of primary human progenitor cells, immune modulation in vivo, and vascularization upon transplantation 140. The rapidity of alginate hydrogel network growth is ideal for constructing complicated and precise 3D bioprinted structures. Singh et al. 58 mixed alginate with kidney-derived dECM to recapitulate the native renal microenvironment. The hybrid bioink underwent rapid cross-linking (**Figure 7B**). Gao et al. 142 produced a bioink consisting of alginate and vascular tissue-derived dECM (VdECM). In addition to facilitating the coaxial printing of vessel-like structures, adding alginate to the synthesized vascular tissue-specific bioink preserved VdECM's ability to stimulate cellular activity. Through the shell and core of a coaxial nozzle, the same group 59 simultaneously extruded a HUVEC-encapsulated VdECM/alginate hybrid bioink and fugitive Pluronic F127 containing Ca2+ ions (CPF127) (**Figure 7C**). The CPF127 solution permitted the ionic gelation of alginate by releasing Ca2+ ions 143. To stabilize the structures, heat cross-linking of the dECM was used to incubate the construct. Two-step ionic/thermal cross-linking enhanced the printability and shape integrity of the constructs. Furthermore, an atherosclerotic model was also developed in vitro using in-bath coaxial cell printing 144. Before printing, CPF127 was injected into the core nozzle. HUVECs were encapsulated using a pH-neutralized hybrid bioink composed of VdECM bioink and sodium alginate, which was attached to the middle nozzle. In the generated models, functional vascular tissues that responded to endothelial dysfunction-inducing stimuli were developed. Under physiological conditions, the existence of vascular tissues in the presence of stenotic and tortuous turbulent flows recapitulates hallmark events in early atherosclerosis. These findings suggested that the constructed atherosclerotic model was a promising platform for atherosclerosis research. In another study, endothelial progenitor cells and atorvastatin-loaded poly (lactic-co-glycolic acid) microspheres were encapsulated in a VdECM-based hybrid bioink and extruded using 3D coaxial cell-printing technology 145 (**Figure 7D**). CPF-127 components were extruded into the inner layer during the printing process, whereas VdECM/alginate comprised the outer layer. Ionic cross-linking of alginate was initiated to ensure initial form fidelity. This cell/drug co-loaded vessel design resulted in exceptional neo-vessel development and limb salvage at 28 days after implantation in the ischemic limbs of mice, demonstrating VdECM's potential to induce neovascularization. Moreover, the presence of alginate could inhibit the rapid breakdown of dECM by native matrix metalloproteinases by crosslinking 146, 147. Unfortunately, the lack of enzymes that can degrade alginate in humans leads to the accumulation of residues in the body, limiting cell function 148. Even though alginate may be easily crosslinked into hydrogels by divalent cations, due to the absence of adhesive sites in cells, alginate must be coupled with other biomaterials. Furthermore, for alginate to undergo ionic cross-linking, the ion concentration must be sufficient to polymerize the alginate components completely. However, overly high ion concentrations may result in cell death due to altered osmotic pressure 144.

## Applications of dECM bioink

**Figure 8:**
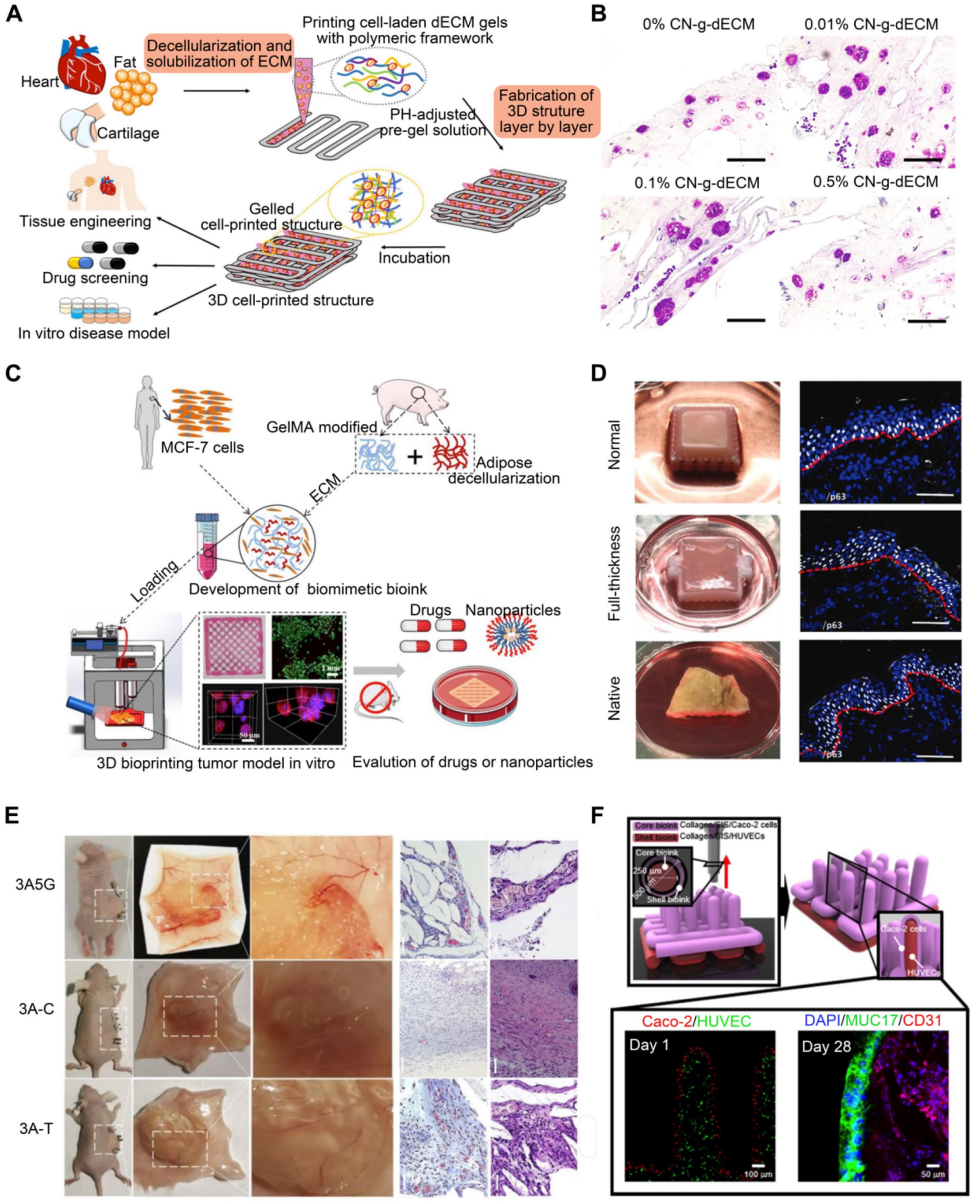
Applications of dECM bioink. (A) The 3D-printed dECM structure has applications in a variety of fields, including tissue engineering, in vitro drug screening, and modeling of tissues and cancer (adapted with permission from 22, copyright 2020 Springer). (B) Increasing the density of the ECM with cellulose nanoparticles modifies the aggressive behavior of gastric cancer cells (adapted with permission from 153, copyright 2021 Elsevier). (C) Schematic illustration of a 3D tumor model bioprinted with ECM-enhanced hybrid bioinks (adapted with permission from 155, copyright 2022 Wiley). (D) A 3D skin equivalent that is perfusable and vascularized, consisting of hypodermis, dermis, and epidermis (adapted with permission from 158, copyright 2019 Wiley). (E) A schematic depiction of the entire procedure for creating the scar model (adapted with permission from 159, copyright 2022 Elsevier). (F) Schematic illustrating the 3D printing method for creating 3D intestinal models with epithelium and capillaries using collagen/SIS bioinks (adapted with permission from 160, copyright 2020 Ivyspring International).

In the past decade, the field of dECM biomaterials has significantly expanded. Applications have branched out from wound healing patches to scaffolds that match physicochemical properties to recreate entire organs. This extends to the recent inclusion of dECM biomaterial applications to the tumor, skin, reproductive tissues, etc. 5, 149. Researchers have realized the enormous potential of dECM biomaterials to mimic the characteristics of biological tissues 48. In this section, we summarize the applications of dECM-based bioinks in 3DBP, including tissue and organ modeling, tissue repair, and the clinical application of dECM (**Figure 8A**).

### 3D printed in vitro tissue or organ models

In the realm of TE, 3D cell-printing technologies have been used to generate artificial tissues or organs 120. These systems precisely deposit biomaterials and cells for the formation of mature tissues or organs. Imitating the forms and functions of natural tissues and organs is challenging for 3D cell printing technologies that fabricate artificial tissue and organ structures 22. The development of real biomimetic in vitro systems for the study of complex diseases will boost the validity of laboratory results 22.

Matrix stiffness in the tumor microenvironment is associated with tumor cell behavior regulation. Most tumors are characterized by abnormal ECM deposition and increased stiffness 150. Certain epithelial tumor cells can undergo epithelial-to-mesenchymal transition when matrix stiffness rises 151. dECM preserves not only the biomechanical properties of the initial tumor but also the composition and architecture of the ECM, generating a perfect tumor microenvironment 152. Kim et al. 153 developed a 3D cell-printing-based gastric cancer model by combining gastric tissue-specific bioinks with cellulose nanoparticles (CNs). They examined the impact of this gastric dECM bioink on gastric cancer cell aggressiveness using histological and genetic techniques. They discovered that adding CNs promoted stomach cancer progression by enhancing the mechanical properties of the matrix (**Figure 8B**). In addition, the CNs-enhanced gastric dECM bioink was utilized to print a variety of 3D forms, including stomach rugae. Using dECM produced from tongue tissue, Kort-Mascort et al. 154 established an in vitro model of head and neck squamous cell carcinoma. The composite material promoted cell proliferation and the formation of tumor-like spheroids. This model could be adapted for applications involving healthy or damaged tissue in TE. This system was also used to investigate small-molecule standard-of-care therapies for this disease. As the properties of the source tissue microenvironment are mimicked, creating in vitro models with reinforced dECM offers a realistic system for evaluating malignant neoplastic events in vitro. Chen et al. 155 utilized 3DBP, adipose ECM-enhanced hybrid bioink, and MCF-7 cells to create a reliable tumor model. The tumor model replicated the essential biological characteristics of in vivo tumors, including complex ECM barriers, multicellular interactions, and proliferation gradients. When comparing 3D-printed tumor models with multicellular spheroid formation to 2D-cultivated cells based on protein and gene expression and tumorigenicity both in vitro and in vivo, 3D-printed tumor models more closely resemble real tumors (**Figure 8C**). These models permit in vitro monitoring of the long-term interactions between drug-loaded nanoparticles and tumor tissue. Future research must combine 3D tumor models and vascular arteries to investigate the enhanced permeability and retention effect during tumor-selective medication delivery and metastasis.

The skin is a sophisticated organ that serves as a barrier, regulator, and messenger 156. The majority of skin substitutes have drawbacks like immune rejection, lack of therapeutic efficacy, and simplicity of tissue-engineered structures 157. Therefore, skin substitutes that integrate the skin's structure with numerous cellular phenotypes and replace the skin's full function are needed. Current skin TE research aims to reproduce the epidermal and dermal layers for full-thickness skin replacement 16. Skin dECM has made much progress toward rebuilding functional skin tissue. The biomolecular cues included in the skin dECM have the potential to regenerate the functional characteristics when designed with diverse skin architecture 16. Using 3D cell-printing technology, Kim et al. 158 created a mature, perusable, and vascularized 3D human skin equivalent consisting of the epidermis, dermis, and hypodermis (**Figure 8D**). This skin model was examined using functional markers for each region to confirm tissue maturity (epidermis, dermis, and hypodermis). Full-thickness skin models more closely resemble natural human skin than do dermal and epidermal skin models. The manufactured skin model has a microenvironment that is more similar to that of native skin than standard skin equivalents, resulting in a more reliable and predictive platform for aesthetic testing, drug screening, and fundamental research. Bin et al. 159 developed a functional human hypertrophic scar model using premade cellular aggregates and dECM-based bioink printing (**Figure 8E**). Firstly, the bioink was created using scar ECM and alginate-gelatin hydrogels with the appropriate physical qualities to imitate the microenvironmental variables; secondly, patient-derived fibroblasts were precultured in the bioink to generate topographic cellular aggregates for future printing. Notably, these scar models represent the earliest stage of scar formation based on gene and protein expression, with the activation of inflammation- and cell-proliferation-related signaling pathways, thus mimicking the in vivo tissue dynamics of scar formation. These models can be employed for precise drug screening as well as for engraftment. In vitro and in vivo models were used to investigate the clinically observed effects of concurrent anti-scarring drug treatment. In addition, the use of materials with differing stiffnesses facilitated the spatial tissue organization of scar collagen bundles in the model. In conclusion, the scar model replicated both biochemical and biophysical characteristics, and its gene expression profile closely resembled that of scar tissue.

Furthermore, Kim et al. 160 created a human intestinal villus model with a novel bioprinting technique employing a collagen/intestinal submucosa (SIS) cell-rich bioink (**Figure 8F**). The collagen/SIS villi contained epithelial cells and demonstrated various cellular activities, including considerable cell proliferation. Collagen/SIS bioink and cell-printing technology may soon be used to develop intestinal models that resemble the human intestine. Compared to the pure cell-laden collagen villus structure with comparable villus geometry, the cell-laden collagen/dECM villus structure offers a more functional intestine-like epithelium. Based on these findings, dECM-based 3D villus models will improve the accuracy of small intestine physiological models.

### 3D printed in vivo tissue repair

Patients with vaginal loss suffer psychological and physical pain; thus, vaginal reconstruction techniques are urgently needed. Traditional TE technology, which uses cells and biomaterials to construct a tissue-engineered vagina, has some efficacy in vaginal reconstruction 161 but has several drawbacks, including low cell survival rates, rough construction, and a lack of personalization. 3DBP technology, a fast-evolving advanced biofabrication technique, could circumvent these issues 162. For instance, Hou et al. 163 used decellularized vaginal matrix bioink to print biomimetic 3D vaginal tissue to restore vaginal morphology and function. In vivo, the bone marrow-derived mesenchymal stem cells-containing 3D scaffold showed an apparent epithelial cell layer, demonstrating that biomimetic 3D vaginal tissues could support epithelialization; therefore, they have great potential for vaginal reconstruction.

Frequently, VML is far too substantial for normal healing, resulting in functional deficits and scar formation 164. Recent research demonstrated that dECM scaffolds can be utilized to facilitate muscle tissue regeneration by modifying the immune response 165. In one study, Choi et al. 166 generated a bioink using porcine SM dECM and C2C12 myoblasts for use with a 3D cell printing method. The dECM bioinks produced from porcine SM offered a myogenic environment for myoblasts, which responded favorably to electrical stimulation. This allowed high cellular contractility and viability, which facilitated the development and maturation of myotubes. This bioink could be used to fabricate functional SM constructs consisting of multinucleated and aligned muscle fibers to replace the native SM. Behre et al. 73 developed a high-concentration bioink composed of dECM and type I collagen and utilized FRESH 3DBP and computed tomography imaging to create patient-specific, large dECM patches for implantation into canine VML wound models. The dimensions of these dECM patches were precise, and they conformed to the surface of complex wounds. Based on 3D imaging data from patients with clinical wounds, these patches can provide patient-specific treatment for soft tissue deficiencies caused by trauma, tumor resection, and other surgical procedures. The purpose of future research should be to determine how specific scaffold microstructure designs, dECM tissue sources, protein compositions, and mechanical properties affect healing processes and functional outcomes.

### dECM for tissue repair in clinical application

ECM derived from tissues or whole organs possesses regenerative benefits. dECM products are gaining clinical significance and market share as a result of their consistent availability for grafting and superiority over competing options, producing greater clinical outcomes than autografts in some applications. The current shortcomings of conventional treatments can be addressed by using dECM. dECM materials have been approved for the treatment of tissue and organ abnormalities and disorders, including orthopedic and dental, cardiovascular, plastic, and reconstructive surgery 46. Several companies are dominating the market with adaptable, decellularized solutions designed for tissue and organ repair. Urinary bladder matrices and SIS are common dECM materials that have been approved by the Food and Drug Administration (FDA) for use in the manufacture of regenerative biomaterials fabrication and can help repair damaged skin, muscle, and gastrointestinal tissues 167. Even though early-stage investigations of organ decellularization are promising, the functional complexity of organs poses a formidable obstacle. Despite early successes, this technology is not yet common practice.

While the skin has a robust wound healing system, extreme injuries such as full-thickness third- or fourth-degree burns or diabetic wounds exceed the skin's regenerative capacity 168. Standard treatment consists of skin autografts, which are ineffective for diabetic wounds and significant burns due to the lack of available skin. Several approved treatments demonstrate the usefulness of skin dECM biomaterials in skin regeneration. The widespread use of decellularized grafts has enhanced diabetic wound healing and increased patient survival for third-degree burns 16. dECM products can promote fibroblast and keratinocyte migration and adhesion, and their growth factors and cytokines can support neovascularization and remodeling, making them the best skin transplantation option. Many decellularized products, such as AlloDerm^®^ regenerative tissue matrix, have been converted for clinical application 46. Following implantation, scar quality and skin function at the implantation site were enhanced 169. Oasis^®^ is another decellularized product for skin repair, derived from decellularized porcine SIS, and primarily used for chronic wound treatment 170. Although there are numerous choices for decellularized skin grafts, none of the available treatments provide scarless healing and the complete creation of skin appendages. Some techniques must be optimized. For instance, clinical products have not demonstrated the reconstitution of adnexal structures such as hair follicles and sweat glands, a crucial criterion for skin development. Fortunately, decellularized human placenta-derived ECM can affect the healing of full-thickness wounds accompanied by hair follicle growth 171. The use of these products has in turn stimulated the growth of decellularization technology. Unfortunately, currently available dermal replacements frequently necessitate a second operation to replace the epidermis.

Tendons and ligaments transmit tension forces between SMs and bones or between bones. Their fundamental purpose is to improve mobility and joint stability 172. The natural structure of the ECM is susceptible to degenerative damage and/or traumas, which may result in a substantial decline. Similar to other tissues, the use of autologous, allologous, or decellularized grafts is favored in situations where self-repair is insufficient 173. These grafts are beneficial for rotator cuff injuries, particularly recurrent cases. Owing to the inadequacy of existing treatments, surgeons are investigating alternative methods for the repair of significant or chronic rotator cuff tears. dECM-based materials are attracting orthopedists' interest. These materials can provide temporary mechanical support and hasten repair with their potential to drive the proliferation and migration of associated cell types. These grafts can be utilized for tendon augmentations, transplantation-based repair, and interpositional arthroplasty 174. Products used for tendon and ligament damage include GraftJacket^®^ and Allopatch HD™. In particular, GraftJacket^®^, created using human dermal dECM, has demonstrated efficacy in the treatment of rotator cuff lesions and rotator cuff rupture and was approved for clinical use in 2014 by the FDA 174. Allopatch HD™ is another commonly used decellularized human dermal product. The efficacy of this product has been studied on high-radius and huge rotator cuff tears with a previous repair history and provides functional tendon repair 175.

Although the decellularization and in vitro recellularization of numerous human tissues and organs have produced good outcomes in studies, there is still a great deal of work to be done. Efforts to construct human grafts of complicated organs (liver, kidney, or lung) are exciting, as their complexity originates from tissue architectures that are difficult to achieve in vitro. The literature reports few attempts to produce bioinks originating from humans 22, but these may be considered landmark studies. Matrigel^TM^ is a commercially available dECM bioink made from a mouse sarcoma cell line and sold as a cell culture supplement. The complex composition of Matrigel^TM^, which mimics the properties of natural ECM, is one of its chief advantages 176. The disadvantage of dECMs, including Matrigel^TM^, is that they are neither quantitatively nor qualitatively defined, and their variability across batches is substantial. Matrigel^TM^ remains a support for bioassays despite its effectiveness for cell culture, but it is improbable that such a substance will ever be put into humans because of safety concerns (i.e., infection and immunogenicity) 75. In addition, as the performance of bioink improves, the promise of making customized 3D reconstructions of patient tissue will further propel bioprinting from the laboratory to the bedside. Bioprinted scaffolds will be difficult to manage from a regulatory standpoint, as they can simultaneously be biologics, pharmaceuticals, and medical devices. Thankfully, regulatory bodies have typically been proactive in offering guidelines, as they acknowledge that it is necessary to move 3DBP closer to clinical use to save lives. Enhancing the in vitro engineering of ECM and encouraging the development of innovative devices will improve personalized therapies. Existing constraints regarding biodevices include the novelty of this sector and the lack of regulatory experience among the small and medium-sized businesses, universities, and academies that develop them. Such products must demonstrate their safety, and only the availability of licenses permits the preclinical examination of human-derived bioengineered products.

In summary, grafting is a viable alternative in situations where other therapeutic options may fail to address tissue or organ damage. Autografts are the first choice, but size constraints and the complications associated with grafts encourage alternate options. Decellularized materials have the capacity to meet the standards afforded by autografts, explaining their wide range of applications. After decellularization, allogeneic or xenogeneic tissues retain their tissue-specific features and bioactive chemicals, depending on tissue type. Hence, dECM materials have the potential to assist tissue regeneration and remodeling. Xenogeneic tissue is a practically limitless source, but it is controversial due to the risk of disease transmission resulting from a failure to optimize decellularization or from low-quality donor animals. While the number of decellularized tissue types accessible for clinical application is limited, the increasing number of pre-clinical and clinical investigations is an encouraging sign. Tissue-specific decellularization is the research ground for the future development of whole-organ decellularization and the manufacture of functional organs for regenerative medicine.

## Perspective and challenges

In the past decade, 3DBP has become increasingly complex and has been investigated for TE and regeneration 65. Many 3D tissue or organ bioprinting strategies have been documented, and certain bioprinted human anatomical parts are already in clinical use 48. With the advancement of material science and the manufacturing industry, additional 3D tissue and organ bioprinting research will focus on optimizing function and building standard systems. dECM bioinks were developed to imitate the structural and functional heterogeneity of native tissues 177, and 3DBP will bridge the gap of dECM between the lab and clinical translation, despite obstacles such as impaired biocompatibility, limited mechanical strength, and insufficient vascularization.

First, the biocompatibility and mechanical strength of bioinks are compromised by printability concerns. Optimal bioinks should be printable, bioactive, biodegradable, stable, reasonably priced, commercially available, and regulated for clinical usage 178. In addition, the bioink's porosity and shape must be suitable for the transfer of cells, gases, metabolites, nutrients, and signal molecules both inside the biomaterial and between the biomaterial and the local environment 179. For instance, dECM possesses ideal biocompatibility but poor extrusion properties and mechanical strength, which can be improved by combining it with printed biomaterials with lower biocompatibility, such as gelatin and sodium alginate 58. However, it is still difficult to find the ideal biomaterial or mix of biomaterials with both promising printability and compatibility with 3DBP, though hybrid hydrogels are by far the most promising route forward. In addition, in situ cross-linkable bioinks with geographically and temporally tunable crosslinking rates and degrees are an intriguing future direction. When creating organ models in vitro, the biomimetic and spatiotemporal requirements of cells, bioink, and the bioprinting process must be considered. Bioinks play a crucial role in 3DBP in simulating the physiological and pathological environment. According to the growth needs of cells in the printed structure, responsive bioink matrix materials are urgently needed to adapt to the dynamic process of tissue development 180. However, synthesizing the optimal bioink with the proper rigidity and cell microenvironment remains difficult 181.

The incorporation of living cells in the manufacturing process makes bioprinting unique. The significance of safeguarding cells has been recognized. In order to maintain structural fidelity and support the subsequent stable culture, the printed structure needs to have a certain mechanical strength (the Young's modulus of the printed hydrogel material is generally 10 kPa or above). However, for many types of soft tissue (the Young's modulus of brain tissue is 1 kPa)-sourced cells, the excellent rigid hydrogels will limit their growth and functional expression 181. Even skeletal TE may require a soft milieu to commence tissue development. 3DBP has been exposed to compromises between physicochemical and biological consequences for a long time. Malda and colleagues coined the term "biofabrication window" to characterize the trade-off for general biofabrication, defining it as "the range of material qualities acceptable for printability with high shape fidelity and for the support of cell function" 21. A bioink with higher viscosity is stiffer, but it may be harmful to cells. Reduced viscosity is more hospitable to cells but hinders the formation of solid structures. Viscosity balance is a crucial part of the bioink preparation process. Among the several methods used to modify the rheological properties and improve printability, adjusting the ratios of different biomaterials is the most prevalent. Combined, structural stability and cell activity requirements have traditionally resulted in a biofabrication window with moderate-strength bioprints. These contradictory requirements drive research towards more complex hydrogel architectures and present intriguing questions about bioink reinforcement efficiency. Novel methods are being explored to expand the biofabrication window by efficiently fortifying hydrogels while preserving their beneficial features. Polymer functionalization, interpenetrating networks, nanocomposites, supramolecular bioinks, and thermoplastic reinforcement are the most prevalent approaches 182, 183. Considering the secretion and remodeling of matrices during tissue creation, the designed ECM does not need to match the mechanical properties of the developed native tissue in the setting of 3D cell culture. To launch the proper cellular processes, however, it is essential to create a sufficient initial mechanical microenvironment.

The reason for using dECM is that it has various advantages, but only considering printability is not enough. For a specific cell, increasing the strength of the bioink rather causes a problem with viability, and problems such as hypoxia occur when manufacturing in large volumes. To precisely imitate biological tissues, bioink should be deposited at a cell-size resolution (510 μm). For clinical applications, thick, multi-layered tissue is necessary. 100-200 μm is the maximal nutrient/oxygen diffusion distance for cells to survive in the absence of vascularization 180. Because of the low viscosity and poor printability of the dECM, it is difficult to extrude a thin print filament that provides an ideal oxygen supply; this will aggravate cell hypoxia. However, increasing the printability of the bioink will harm cell viability due to its increased viscosity. One way to solve this contradictory problem is to find appropriate vascularization procedures. However, bioprinted dECM tissue substitutes lack effective vascularization procedures. It remains difficult to build regulated networks resembling vascular trees. The realization of vascularized TE may be an obstacle for the following decade. In recent decades, the structural complexity of TE bioprinting techniques has increased dramatically, but bioprinting of soft materials (e.g., hydrogels) is still immature and numerous obstacles remain 184.

Another trend in TE research is the integration of 3DBP with other biofabrication methods, seeking to capitalize on their respective strengths. Other new technologies, such as four-dimensional (4D) bioprinting 185, bioprinted organs-on-a-chip 186, and microfluidics-assisted extrusion bioprinting 187, are also promising. Derived from 3DBP, 4D bioprinting may potentially recreate the spatiotemporal changes in tissue geometry and the spatial distribution transformations of cells and ECM. For example, Wang et al. 186 introduced a novel 3D tumor progression model based on metastasis-on-a-chip with organ-specific ECM to predict treatment success. The kidney cancer cells were cultivated in a liver ECM to recreate the liver milieu, predict therapeutic effects, and evaluate dose response at various stages of tumor progression. The tumor progression model based on metastasis-on-a-chip and organ-specific ECM is a powerful tool for swiftly analyzing treatment regimens and designing chemotherapeutic drugs. Dickman et al. 187 created a novel microfluidic 3DBP technology to generate live and functioning smooth muscle tissue by incorporating dECM into 3D cell culture.

Conventional decellularization techniques usually produce dECM with batch-to-batch variance, unstable gelation, and changed composition, including the loss of GAGs and growth factors 12. In addition, because dECM is derived from different tissues, it offers a higher range of structural, chemical, and biological cues than other biomaterials 23, 188. For future in vivo follow-up studies, the quality control of dECM should be standardized according to the ECM source or organ/tissue type. The elimination of cellular components and antigens from the native ECM minimizes the likelihood of unfavorable effects at the graft site, such as an inflammatory response and immune rejection. Because the cells are typically linked to or encased within the ECM, it is hard to eradicate all antigens from the construct without compromising ECM integrity. However, numerous clinical products contain cell fragments and cellular DNA. These cell and DNA remnants trigger pro-inflammatory responses, which expedite the remodeling and tissue repair processes. Although the actual process remains ambiguous 189, constructions with high bioactive ECM components and optimum quantities of antigens can help regulate cellular behavior in clinical settings 190. Balancing the amount of cell fragments necessary to sustain bioactivity but not enough to elicit an immune response will require research. In addition, while selecting composite materials, it is essential to note that polymers and crosslinking may influence the immune cell response. Degradation byproducts of polymers such as PEG and crosslinkers may be cytotoxic, leading to a rise in inflammation once again. Integrating material-driven immunomodulatory methods is therefore crucial.

Quantification of endotoxin contamination is also essential for assessing the appropriateness of dECM for cellular encapsulation, as endotoxin contamination can result in increased islet inflammatory cytokine production 191. The FDA specifies the endotoxin contamination limit for medical devices or drugs at 0.5 EU/mL; nevertheless, extraction and elution screens do not fully remove contamination due to endotoxin's tendency to adhere to material surfaces 192. Concerningly, contamination levels in lung dECM gels were over sevenfold higher than FDA recommendations for extracts and up to sixteenfold higher than those reported for other tissue sources. While additional cleaning by terminal sterilization (e.g., gamma irradiation and ethylene oxide) could lessen this contamination, the resulting gels are likely to have diminished mechanical and biochemical integrity as well as decreased cellular adhesion 193. This tissue source diversity, despite the use of aseptic methods and antibiotics during decellularization, highlights the need for quantification of endotoxin contamination for all ECM studies.

## Conclusion

dECM is a promising material for TE. Toxicity, mechanical properties, and immune-related issues are the greatest challenges for dECM-based approaches. Particularly, additional research is needed on achieving the balance between its biological and mechanical properties. Furthermore, studies have explored various physical and chemical approaches to enhance the printability of dECM, each with its boundedness. For the industrialization of dECM bioink bioprinting, factors beyond tissue or organ selection must be considered, such as the decellularization process, sterilization, cost, yield, quality, and batch variance of dECM bioinks. Without a multidisciplinary design incorporating cell biology, material science, physics, and mechanical engineering, the clinical utility of dECM cannot be achieved. In the future, we hope to optimize the decellularization process and develop more standardized decellularization evaluation criteria to improve the quality, quantity, and reproducibility of dECM; develop dECM-based bioinks with controlled printability, degradation, and biological properties; construct tissues and organs using 3DBP technology; and create molecularly engineered dECM.

## Figures and Tables

**Table 1:** Examples of materials for improving dECM-based bioink printability

Category	Materials	Usage	Strategy	Applications	Bioprinting Strategy	References
Synthetic polymers	PCL	Long-term supportive materials	Physical strategy	Tissue-derived dECM-based bioinks to manufacture 3D tissue constructions	Extrusion Printing	22
	PCL	Long-term supportive materials	Physical strategy	A 3D cell-printed dome-shaped construct to reconstruct the breast.	Extrusion Printing	55
	PCL	Long-term supportive materials	Physical strategy	A functioning in vitro airway-on-a-chip connected to a vascular network	Extrusion Printing	77
	PEVA	Long-term supportive materials	Physical strategy	An engineering heart tissue model.	Extrusion Printing	56
Natural polymers	Hyaluronic acid methacrylate	Cross-linkable hydrogel	Chemical strategy	A 3D-printed islet organoid.	Digital light processing printing	124
	Thiol-functionalized hyaluronic acid	Cross-linkable hydrogel	Chemical strategy	A customed scaffold implanting custom scaffolds into an articular cartilage defect	Extrusion Printing	127
	Alginate	Cross-linkable hydrogel	Chemical strategy	Perfusable renal proximal tubule and blood vessel structures	Extrusion Printing	58
	Alginate	Cross-linkable hydrogel	Chemical strategy	facilitating the coaxial printing of vessel-like structures	Extrusion Printing	142
	Alginate	Cross-linkable hydrogel	Chemical strategy	A vessel structure to deliver endothelial progenitor cells and the proangiogenic drug Atorvastatin for the treatment of ischemic diseases	Extrusion Printing	145
	GelMA	Cross-linkable hydrogel	Chemical strategy	Pattern liver dECM with tailorable mechanical properties	Digital light processing printing	97
	GelMA	Cross-linkable hydrogel	Chemical strategy	A 3D-printed heart structure	Digital light processing printing	115
	GelMA	Cross-linkable hydrogel	Chemical strategy	Fabricate an inner gear-like structure of liver microtissue.	Digital light processing printing	98
	GelMA	Cross-linkable hydrogel	Chemical strategy	A biomimetic scaffold similar to the native meniscus	Extrusion Printing	117
Photo-initiator	Ruthenium/Sodium persulfate	Initiate photo-crosslinking	Chemical strategy	Fabrication of complicated constructs with high aspect ratios	Digital light processing printing	102
	Ruthenium/Sodium persulfate	Initiate photo-crosslinking	Chemical strategy	A perfusable tubular model	Digital light processing printing	133
	Vitamin B2	Initiate photo-crosslinking	Chemical strategy	Improved mechanical properties of heart constructs	Digital light processing printing	18
	PEGDA	Photo crosslinking agent	Chemical strategy	A bioprinted liver construct	Digital light processing printing	17
	PEGDA	Photo crosslinking agent	Chemical strategy	Mimic both normal and fibrotic cardiac tissues simply by controlling the mechanical properties.	Digital light processing printing	136
Sacrificial materials	Pluronic F-127	Temporary supportive materials	Physical strategy	The orientation and stable geometric structure of in vitro-grown biliary trees	Extrusion Printing	85
	Gelatin granule	Temporary supportive materials	Physical strategy	Freestanding multilayered dECM-based tendon/ligament structures	Extrusion Printing	88
	Gelatin granule	Temporary supportive materials	Physical strategy	Hierarchical architecture of vascularized muscle	Extrusion Printing	95
	Gelatin microparticle	Temporary supportive materials	Physical strategy	Three-dimensional printing of complex biological structures by freeform reversible embedding of suspended hydrogels	Extrusion Printing	73
	Agarose microparticle	Temporary supportive materials	Physical strategy	Bioinks that promote renal growth and differentiation of reparative human renal progenitor cells.	Extrusion Printing	92
Functionalized with methacrylate groups	Methacrylated kidney dECM	Cross-linkable hydrogel	Chemical strategy	Functional kidney microtissues in vitro	Extrusion Printing	61
	Methacrylated porcine skeletal muscle-derived dECM	Cross-linkable hydrogel	Chemical strategy	Skeletal muscle-like tissue constructs with biochemical and topographical cues	Extrusion Printing	62
	Methacrylated cartilage-derived ECM	cross-linkable hydrogel	Chemical strategy	A printed anatomical ear shape	Extrusion Printing	110

## Abbreviations

ECM: extracellular matrix
dECM: decellularized ECM
TE: tissue engineering
3DBP: 3D bioprinting
GAGs: glycosaminoglycans
H&E: hematoxylin and eosin
DAPI: 4',6-diamidino-2-phenylindole
DLP: digital light processing
PCL: polycaprolactone
PEVA: poly (ethylene/vinyl acetate)
GelMA: gelatin methacrylate
PEG: polyethylene glycol
FRESH: freeform reversible embedding of suspended hydrogels
SM: skeletal muscle
PVA: polyvinyl alcohol
VML: volumetric muscle loss
HUVECs: human umbilical vein endothelial cells
UV: ultraviolet
dERS: ruthenium/sodium persulfate
HAMA: hyaluronic acid methacrylate
PEGDA: polyethylene glycol diacrylate
Hon: honokiol
VdECM: vascular tissue-derived dECM
CPF127: pluronic F127 containing Ca2+ ions
CNs: cellulose nanoparticles
SIS: intestinal submucosa
FDA: Food and Drug Administration
4D: four-dimensional
